# Fast and Inexpensive Detection of Total and Extractable Element Concentrations in Aquatic Sediments Using Near-Infrared Reflectance Spectroscopy **(**NIRS**)**

**DOI:** 10.1371/journal.pone.0070517

**Published:** 2013-07-29

**Authors:** Till Kleinebecker, Moni D. M. Poelen, Alfons J. P. Smolders, Leon P. M. Lamers, Norbert Hölzel

**Affiliations:** 1 Institute of Landscape Ecology, University of Münster, Münster, Germany; 2 B-Ware Research Centre, Nijmegen, The Netherlands; 3 Department of Aquatic Ecology and Environmental Biology, Radboud University Nijmegen, Nijmegen, The Netherlands; National Research Council of Italy, Italy

## Abstract

Adequate biogeochemical characterization and monitoring of aquatic ecosystems, both for scientific purposes and for water management, pose high demands on spatial and temporal replication of chemical analyses. Near-infrared reflectance spectroscopy (NIRS) may offer a rapid, low-cost and reproducible alternative to standard analytical sample processing (digestion or extraction) and measuring techniques used for the chemical characterization of aquatic sediments. We analyzed a total of 191 sediment samples for total and NaCl-extractable concentrations of Al, Ca, Fe, K, Mg, Mn, N, Na, P, S, Si, and Zn as well as oxalate- extractable concentrations of Al, Fe, Mn and P. Based on the NIR spectral data and the reference values, calibration models for the prediction of element concentrations in unknown samples were developed and tested with an external validation procedure. Except Mn, all prediction models of total element concentrations were found to be acceptable to excellent (ratio of performance deviation: RPD 1.8–3.1). For extractable element fractions, viable model precision could be achieved for NaCl-extractable Ca, K, Mg, NH_4_^+^-N, S and Si (RPD 1.7–2.2) and oxalate-extractable Al, Fe and P (RPD 1.9–2.3). For those elements that showed maximum total values below 3 g kg^−1^ prediction models were found to become increasingly critical (RPD <2.0). Low concentrations also limited the performance of NIRS calibrations for extracted elements, with critical concentration thresholds <0.1 g kg^−1^ and 3.3 g kg^−1^ for NaCl and oxalate extractions, respectively. Thus, reliable NIRS measurements of trace metals are restricted to sediments with high metal content. Nevertheless, we demonstrated the suitability of NIRS measurements to determine a large array of chemical properties of aquatic sediments. The results indicate great potential of this fast technique as an analytical tool to better understand the large spatial and temporal variation of sediment characteristics in an economically viable way.

## Introduction

Soils and sediments of natural systems not only show high spatial and temporal variation in physical and chemical characteristics, but also in fluxes of elements among this compartment, the hydrosphere and the atmosphere [1], [2]. Given the importance of soils and sediments, e.g. with respect to their functioning as a fundamental nutrient resource for primary production (including crop production) and their regulatory and filtering services for surface water and groundwater, there is a strong need for spatial mapping and regular monitoring in order to be able to detect patterns and changes in biogeochemical characteristics of soils and sediments [3], [4], [5]. Particularly in aquatic ecosystems and wetlands, information on the distribution and retention of elements in the sediments is of crucial importance because of the strong interactions between the sediment and the surface water with respect to nutrients and contaminants [6], [7], [8].

Aquatic sediments can act both as a source and as a sink from which element ions and charged molecules can be released or immobilized. Thus, sediment characteristics provide indispensable information for the understanding of biogeochemical key processes in aquatic systems and wetlands related to major issues such as eutrophication and toxicity [9], [10], and can therefore be used for the prediction of the fast biotic response to environmental change (within a timeframe of days to weeks), e.g. after restoration measures have been taken [8], [11]. One of the biogeochemical key processes causing massive eutrophication is sulfate-mediated phosphate mobilization in the sediment [10], [12]. In this context, knowledge about sediment phosphorus, sulfur and iron contents including differently extracted fractions add particularly valuable information for process understanding and the selection of appropriate measures to counteract massive sulfate-mediated phosphate release. Geurts et al. [8] for instance showed that the iron to sulfur ratio of the bulk sediment was a strong indicator for the release of phosphorus to the surface water. Next to determining nutrient release or immobilization rates, the chemical characteristics of sediments strongly affect the sorption of contaminants [13] and the degree of acidification and concomitant release of metals upon desiccation [14]. Therefore, the element composition of sediments provides important information about their biogeochemical role in aquatic systems.

These examples demonstrate the importance of a comprehensive chemical characterization of sediments in aquatic systems, both for scientific and for applied issues including water management. However, an integrated assessment of the spatial distribution of sediment quality data is often lacking [3], [5], most likely due to financial constraints as sample processing (digestion and extraction methods) and chemical analyses of large numbers of samples and their different chemical properties are costly and time consuming. Moreover, seasonal variability may additionally affect element exchange rates between sediment and water and add a temporal dimension to these environmentally relevant processes [2]. Thus, a rapid, inexpensive and reproducible method for the determination of various chemical properties of sediments would be very useful for many environmental studies and monitoring programs related to aquatic systems and wetlands.

Near-infrared reflectance spectroscopy (NIRS), being a fast and low-cost analytical technique, has this potential to offer a reliable alternative to traditional chemical analyses, without the need of elaborate and costly sample preparation including microwave digestion or extractions with various solvents [15]. Near infrared analysis is an indirect method that estimates the chemical component of interest by linking spectral characteristics to the known chemical composition. The analyses of unknown samples can subsequently be carried out with a calibration model that has to be developed using multivariate regression procedures [16]. NIRS is routinely used for the analysis and quality control of pharmaceutical and agricultural products [17], [18]. Recently, the great potential of this technique for environmental and soil ecological studies has increasingly been recognized [19], [20], [21], [22], [23], [24]. However, although research on NIR/MIR spectroscopy for soil analysis has experienced a boom over the last 10 years [25], the NIRS technology is still relatively unpopular in soil science, probably due to the fact that the method is largely unknown to a considerable part of the scientific community concerned [26].

Near infrared spectroscopy is primarily based on absorbance characteristics caused by vibrations of covalent bonds between H, C, O and N, which are the main constituents of organic matter [27]. Therefore, it was proven to be a feasible method for the substitute measurement of organic soil constituents such as C and N contents or of more complex substance classes such as fibres and phenolics [28], [29]. Although metals and other mineral compounds such as phosphorus do not absorb radiation in the NIR region they may still be detectable via their association with organic matter, oxides, hydroxides or clays that absorb light in the NIR wavelength range [26], [30]. However, for the measurement of various total and exchangeable chemical substances NIRS calibration models have been shown to perform with great variability among different studies [4], [31], [32], showing that additional research is required.

Here, we present NIRS calibration models for the measurement of various chemical properties of aquatic sediments sampled at 191 locations across The Netherlands. These samples showed a strong variation in organic matter content and in concentrations of target variables, which is vital for a reliable study. Our analyses included 1) total element concentrations of Al, C, Ca, Fe, K, Mg, Mn, N, Na, P, S, Si and Zn, 2) NaCl-extractable concentrations of Al, Ca, Fe, K, Mg, Mn, NH_4_-N, NO_3_-N, P, S, Si and Zn, and 3) oxalate-extractable Al, Fe, Mn and P. Different prediction models are evaluated and the applicability for fundamental and applied environmental research is discussed.

## Materials and Methods

### Ethic statement

We got permission from in total 11 different waterboards to conduct the study on the 191 different locations. These waterboards gave permission on the following locations:

Hoogheemraadschap van Delfland (location 1–17, 86–99), Waternet (location 18–33, 100–109, 154–158), Hoogheemraadschap van Schieland en Krimpenerwaard (location 34–40), Waterschap Brabantse Delta (location 41–45), Wetterskip Fryslan (location 46–50, 66–75, 129–133), Waterschap Reest en Wieden (location 51–55), Waterschap de Veluwe (location 56–65, 134–143), Waterschap Hunze en Aa's (location 76–85, 110–114, 159–163), Hoogheem raadschap van Rijnland (location 115–128, 181–192), Hoogheemraadschap de Stichtse Rijnlanden (location 144–153, 171–180), Waterschap Rivierenland (location 164–170).

There was no private land involved, and there were no endangered or protected species involved.

### Sampling and chemical analysis

From 191 locations distributed over The Netherlands sediment samples (0–10 cm) were collected from lakes and ditches using a piston sampler (Eijkelkamp Agrisearch Equipment, Giesbeek, The Netherlands). Samples were gathered during two years: between the 18^th^ of June and the 19^th^of July 2010 and between the 7^th^ of June and the 14^th^ of July 2011. All samples were kept in air tight bags and immediately transported in a cool-box to the laboratory for further analyses.

To determine total element concentrations, sediment samples were dried for 48 h at 60°C and then ground in liquid nitrogen. Total nitrogen and carbon concentrations were measured with a CNS analyzer (type NA1500; CarloErba Instruments, Milan, Italy). Total concentrations of all other elements were considered as the concentration after digestion with HNO_3_ and H_2_O_2_, thus not including the element fraction present in the mineral matrix of particles [9]. Concentrations were determined by digesting 200 mg of dried material in sealed Teflon vessels in a Milestone microwave oven (Ethos D microwave labstation; Milestone S.r.l., Sorisole, Italy) after addition of 4 mL HNO_3_ (65%) and 1 mL H_2_O_2_ (30%). After dilution of the digests, element concentrations were determined by ICP-AES (IRIS Intrepid II; Thermo Electron Corporation, Franklin, MA, USA).

The concentrations of amorphous Fe-, Mn- and Al oxides, which are supposed to represent the amorphous (non-crystalline) fractions of these elements that are able to adsorb P, and the adsorbed P were determined by oxalate extraction: 2.5 g of sediment was shaken (100 rotations per minute) for 2 h with 50 ml of a solution containing ammonium oxalate (115 mmol L^−1^) and oxalic acid (85 mmol L^−1^) [33]. To determine the NaCl-extractable ion concentrations, 17.5 gram of fresh sediment was shaken (100 rotations per minute) for 2 h with an anoxic solution (50 ml) containing 250 mmol L^−1^ NaCl [34]. Element concentrations were determined by ICP-AES (IRIS Intrepid II; Thermo Electron Corporation, Franklin, MA, USA). Concentrations of nitrate and ammonium were measured colorimetrically using a Traacs 800+ auto-analyzer as described by Tomassen et al. [35].

### NIRS analysis

Prior to spectral analyses, air dried samples were screened through a sieve with 2 mm mesh wire. To ensure equal sample moisture, samples were additionally dried for 2 h at 60°C. Subsequently, NIR spectral data was recorded with a Spectra Star 2400 (Unity Scientific, Columbia, Maryland, United States). Each scan consisted of 24 single measurements, which were averaged to one spectrum. Measurements were made at 1 nm intervals over a range of 1250 to 2350 nm. Spectral data were recorded as absorbance (log1 R^−1^, where R = reflectance) and first (1D) derivative of absorbance. For each parameter, the total sample set was divided into two groups: a calibration group and an external validation group. Samples for validation were selected by arranging the respective parameters in ascending order and taking each fifth sample. The remaining samples were used for calibration. This selection procedure ensured the representativeness of both the calibration and the validation set along the range of observed values.

Prior to the calibration procedure, spectra were mathematically corrected for light scattering using the standard normal variate correction. Calibrations were calculated by partial least-squares regression using CalibrationWorkshop (SensoLogic software GmbH, Norderstedt, Germany). After cross validation with 50 segments, the standard error of cross validation (SECV) was used to determine the number of factors used for calibration. This was usually the case when the first minimum of SECV occurred. Calculating calibrations, several samples were identified as outliers. These outliers were characterized by a high residual error compared to reference measurements or spectral dissimilarity to the total data set. Where appropriate, prediction models were optimized by excluding such outliers from calibration. However, in order not to overestimate the applicability of NIRS, the maximum number of outliers removed was set to five.

The optimum calibration equations were selected based on a high coefficient of multiple determinations (R), a low standard error of calibration (SEC) and a low SECV. The SECV is a measure for the difference between the actual and the predicted values calculated over all cross-validation calibrations. The SEC is exclusively based on spectra used for calibration. It indicates the theoretical precision of the calibration to predict samples of unknown composition. The reliabilities of the different models were additionally tested by independent validation samples. The coefficient of determination for prediction (r) and the standard error of prediction (SEP) were used to additionally evaluate the precision of the NIRS model. For evaluation purposes the ratio of performance deviation (RPD  =  standard deviation of reference values/SEP) and the ratio of the interquartile distance of reference values to SEP (RPIQ) were calculated [36].

The final evaluation of the calibration models was based on the suggestions given by Saeys et al. [37]. Prediction models were considered to be excellent when the RPD value and r^2^ (and R^2^) were >3.0 and 0.90, respectively. Values from 2.0 to 3.0 (RPD) and >0.75 (r^2^, R^2^) were indicative for calibration models that allow for good to approximate quantitative predictions. The possibility to distinguish between high and low concentrations within the range of observed values is revealed by values between 1.5 and 2.0 (RPD) and >0.65 (r^2^, R^2^). Unsuccessful predictions have RPD or r^2^/R^2^ values lower than 1.5 or 0.65, respectively.

## Results and Discussion

**Figure 1:**
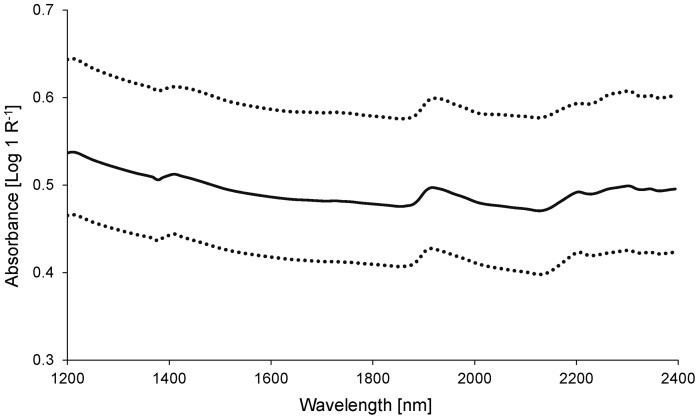
Mean NIR spectral absorbance (Log 1 R^−1^, R = reflectance) of sediment samples. *Solid line* mean spectra of all samples, *upper dashed line* mean spectra of samples with >30% organic matter, *lower dashed line* mean spectra of samples with <30% organic matter.

The mean absorbance values of the analyzed sediment samples revealed three prominent peaks around 1400, 1900, and 2200 nm and small absorption peaks around 2300 and 2350 nm (Fig. 1). The spectral bands found in this study are consistent with data reported in literature, where absorbance around 1400 and 1900 nm was attributed to O–H bonds and absorbance around 2200 nm was found to be related to absorption by C–H bonds [19], [38]. As organic matter and soil minerals have multiple absorption bands >2100 nm, a definite assignment of the two small peaks around 2300 and 2350 nm to specific functional groups is not possible [28], [39]. Both samples with high and with low organic matter content showed the same runs of the curves, but samples with high organic matter content had clearly higher NIR-optical density (see Fig. 1). This was in accordance with the study of Chang and Laird [28], who found weak reflectance (strong absorbance) of dark colored humic acids in the visible and NIR wavelength range between 400 and 2500 nm.

The analyzed sediment samples showed a high variability in the measured chemical soil properties, which was good because this made them representative for a wide range of aquatic sediments (Table 1). Sufficiently high variation in the constituent values is a precondition for the development of feasible NIRS calibrations [30]. This variation particularly includes a wide range in organic matter contents (as determined by loss on ignition) from <0.1 to >85%. Percentage organic matter and total C (and total N) were highly correlated (r = 0.92 [C] and r = 0.88 [N]), indicating that total C of the analyzed samples was almost entirely originating from organic compounds. Except for S (r = 0.48), all other total element concentrations hardly showed any correlation with the organic matter content. Contrary, remarkable correlations were observed between some NaCl-extractable element fractions and the organic matter content (r = 0.82 [Ca], r = 0.55 [K], r = 0.75 [Mg]; Table 2), which may be explained by the important role of organic matter in the cation exchange capacity of sediments.

**Table 1:** Statistical summary of the sample dataset.

	Calibration data set (N = 153)			Validation data set (N = 38)			Total data set (N = 191)		
	Range	Mean	SD	Range	Mean	SD	Range	Mean	SD
Total concentrations [g kg^−1^]									
Al	0.13/16.15	4.51	3.63	0.32/12.61	4.77	3.39	0.13/16.15	4.56	3.57
C	1.6/471.3	128.2	118.3	1.9/449.9	130.8	118.2	1.6/471.3	128.7	117.9
Ca	0.29/106.90	15.48	19.85	0.47/79.99	17.14	19.56	0.29/106.90	15.81	19.75
Fe	0.10/33.61	11.64	8.89	0.80/30.07	12.63	8.32	0.10/33.61	11.84	8.76
K	0.03/3.10	0.79	0.76	0.04/2.48	0.75	0.68	0.03/3.10	0.78	0.74
Mg	0.08/6.85	1.71	1.62	0.10/5.10	1.88	1.57	0.08/6.85	1.74	1.61
Mn	0.01/1.32	0.38	0.38	0.01/1.29	0.38	0.38	0.01/1.32	0.38	0.38
N	0.12/26.70	9.09	7.72	0.35/22.75	9.50	7.78	0.12/26.70	9.17	7.72
Na	0.00/1.12	0.20	0.25	0.00/0.93	0.20	0.22	0.00/1.12	0.20	0.24
P	0.02/2.48	0.65	0.61	0.02/2.30	0.68	0.61	0.02/2.48	0.65	0.61
S	0.24/34.29	6.50	8.58	0.45/32.26	7.09	8.58	0.24/34.29	6.61	8.55
Si	0.02/2.76	0.52	0.58	0.02/2.22	0.53	0.54	0.02/2.76	0.52	0.57
Zn	0.00/1.28	0.10	0.17	0.00/0.67	0.11	0.15	0.00/1.28	0.10	0.17
NaCl-extractable concentrations [mg kg^−1^]; * [g kg^−1^]									
Al	0.00/6.45	0.69	1.03	0.00/3.00	0.67	0.72	0.00/6.45	0.67	0.97
Ca*	0.00/8.98	2.95	2.33	0.12/7.75	2.93	2.27	0.00/8.98	2.92	2.29
Fe	0.00/13.45	0.85	2.21	0.00/9.27	0.67	1.64	0.00/13.45	0.82	2.11
K	0.00/335.46	125.02	90.05	0.00/309.12	122.91	88.02	0.00/335.46	124.60	89.41
Mg*	0.00/1.01	0.31	0.25	0.00/1.94	0.34	0.27	0.00/1.01	0.32	0.26
Mn	0.01/78.00	18.32	18.73	0.03/63.84	17.96	18.47	0.01/78.00	18.24	18.47
N-NH_4_	0.35/369.47	71.90	91.45	1.34/333.92	81.06	96.52	0.35/369.47	73.76	92.30
N-NO_3_	0.04/10.02	1.16	1.70	0.06/7.14	1.19	1.71	0.04/10.02	1.17	1.70
P	0.00/13.73	0.90	2.01	0.02/5.58	0.64	1.00	0.00/13.73	0.84	1.84
S*	0.00/1.55	0.28	0.30	0.01/0.90	0.26	0.26	0.00/1.55	0.27	0.29
Si	0.01/188.45	46.80	45.86	0.71/166.99	46.37	45.90	0.01/188.45	46.71	45.74
Zn	0.00/3.24	0.50	0.57	0.01/2.77	0.51	0.59	0.00/3.24	0.50	0.57
Oxalate-extractable concentrations [g kg^−1^]									
Al	0.10/3.27	1.14	0.81	0.12/3.09	1.19	0.80	0.10/3.27	1.20	1.14
Fe	0.17/58.72	9.55	11.64	0.33/45.35	9.46	10.68	0.17/58.72	9.53	11.42
Mn	0.00/3.34	0.40	0.53	0.01/1.89	0.35	0.36	0.00/3.34	0.39	0.50
P	0.01/2.93	0.64	0.62	0.02/2.19	0.69	0.59	0.01/2.93	0.65	0.61

**Table 2:** Calibration and validation statistics of partial least-squares regression models.

	Transf.	No. factors	R^2^	SEC	SECV	r^2^	SEP	RPD	RPIQ	Corr. OM
Total concentrations [g kg^−1^]										
Al	abs	13	0.86	1.44	1.62	0.84	1.35	2.5	3.8	0.04
C	1D	10	0.89	39.8	48.7	0.85	45.6	2.6	4.0	0.92
Ca	1D	10	0.87	7.55	9.47	0.77	9.45	2.1	2.2	0.01
Fe	1D	11	0.86	3.50	4.25	0.80	3.73	2.2	3.6	0.10
K	1D	9	0.83	0.33	0.37	0.82	0.32	2.2	2.6	0.10
Mg	1D	11	0.87	0.60	0.74	0.81	0.72	2.2	3.6	−0.05
Mn	1D	12	0.69	0.22	0.29	0.45	0.28	1.4	1.5	0.09
N	1D	11	0.93	2.11	2.58	0.91	2.49	3.1	5.6	0.88
Na	1D	4	0.80	0.11	0.12	0.72	0.12	1.9	1.8	0.29
P	abs	9	0.79	0.29	0.32	0.76	0.31	2.0	2.5	0.00
S	1D	11	0.86	3.27	4.08	0.83	3.84	2.2	3.2	0.44
Si	1D	14	0.85	0.24	0.32	0.70	0.30	1.8	2.2	−0.01
Zn	abs	5	0.68	0.10	0.12	0.74	0.09	1.8	1.1	−0.02
NaCl-extractable concentrations [mg kg^−1^]; * [g kg^1^]										
Al	abs	15	0.74	0.49	0.64	0.28	0.79	0.9	1.0	0.30
Ca*	abs	14	0.83	1.02	1.27	0.77	1.14	2.0	3.2	0.82
Fe	abs	15	0.55	1.56	2.04	0.01	2.26	0.7	0.2	−0.03
K	abs	13	0.81	41.56	51.91	0.80	39.67	2.2	3.6	0.55
Mg*	abs	14	0.82	0.11	0.14	0.76	0.13	2.1	3.0	0.75
Mn	abs	8	0.49	13.74	15.10	0.27	15.56	1.1	1.5	0.29
N-NH_4_	1D	4	0.75	48.77	51.38	0.78	47.18	2.1	2.1	0.36
N-NO_3_	1D	8	0.60	1.11	1.31	0.44	1.26	1.4	0.8	0.42
P	abs	9	0.58	1.34	1.54	0.30	1.21	0.8	0.5	0.06
S*	abs	17	0.84	0.13	0.18	0.72	0.15	1.8	2.1	0.41
Si	Abs	14	0.78	22.73	28.55	0.71	26.76	1.7	2.1	0.63
Zn	1D	6	0.45	0.43	0.50	0.31	0.49	1.2	1.2	0.26
Oxalate-extractable concentrations [g kg^−1^]										
Al	abs	13	0.84	0.35	0.41	0.84	0.34	2.3	3.5	0.43
Fe	abs	10	0.86	4.55	5.18	0.82	4.59	2.3	2.2	0.10
Mn	abs	14	0.71	0.30	0.40	0.30	0.39	1.1	1.1	0.11
P	abs	10	0.78	0.30	0.33	0.72	0.31	1.9	2.9	−0.04

Calibrations were evaluated as follows (Saeys et al (2005): excellent (R^2^/r^2^ ≥ 0.9, RPD ≥ 3.0), reliable quantitative predictions (R^2^/r^2^ ≥ 0.75 and <0.9, RPD ≥ 2.0 and <3.0), differentiation between high and low values (R^2^/r^2^ ≥ 0.65 and <0.75, RPD ≥ 1.5 and <2.0, unsuccessful (R^2^/r^2^ <0.65, RPD <1.5).

Transf: transformations for regression analyses.

Abs: Log1/R (R = reflectance).

1D: first derivative.

R^2^: coefficient of multiple determination (calibration).

SEC: standard error of calibration.

SECV: standard error of cross validation.

r^2^: regression coefficient NIRS predicted vs. observed values.

SEP: standard error of prediction (validation).

RPD: ratio of SD of reference values (validation) to SEP.

RPIQ: ratio of the interquartile distance IQ ( = Q3–Q1) of reference values (validation) to SEP.

Corr. OM: Pearson correlation with organic matter content.

### Measurement of total element concentrations

**Figure 2:**
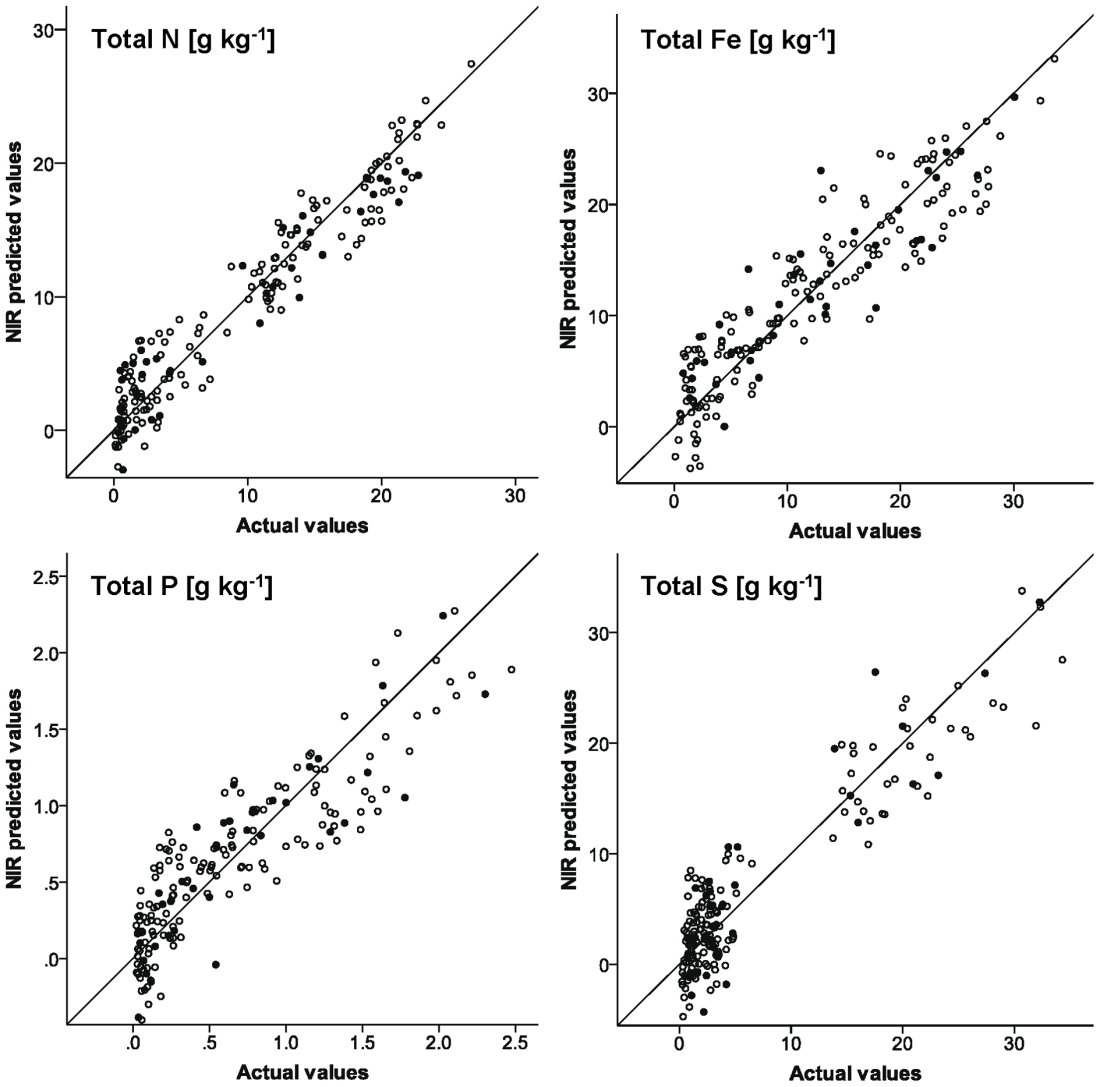
Exemplary observed vs. NIR predicted values for total concentrations of N, Fe, P, and S. *Open circles* calibration data set, *filled circles* external validation.

The calibration for nitrogen was excellent and consistent with the quality reported in literature for NIRS measurements for total N of soils and sediment samples (Fig. 2) [28], [40], [41]. The R^2^ was 0.93 and the SEC as well as the SECV were <2.5 g kg^−1^. The external validation confirmed the high predictive power of the model (RPD: 3.1, see Table 2). Good prediction accuracy was also achieved for total C concentrations, which is in accordance with NIRS calibrations for aquatic sediments presented by others [42], [43], [44]. However, literature also shows examples of poor NIRS prediction model quality for C concentrations in soils, which could mainly be traced back to (1) an insufficient number of samples for calibration and (2) missing spectral representativeness for a defined sample population [26]. Our large sample set covering a broad range of spectral and chemical sediment characteristics demonstrated the general ability of the NIRS technique to accurately measure total C concentrations when these two requirements are met.

The other elements analyzed do not have a primary response in the NIR region, but may be predicted by means of NIRS owing to their correlations with spectrally active constituents [26]. Beside organic matter, sulphate, carbonate and OH groups of soil minerals are reported to have distinct absorption characteristics in the NIR region and may explain successful prediction models of elements without primary response in the NIR region [38]. We were able to obtain calibration models that enable reliable quantitative predictions within the range of observed values for total Al, Ca, Fe, K, Mg, P and S (R^2^ 0.79–0.87, r^2^ 0.76–0.84, RPD 2.0–2.5; see Table 2 and Figure S1). For total Na, Si and Zn concentrations NIRS prediction models were at least able to distinguish between high and low concentrations within the range of observed values (R^2^ 0.68–0.85, r^2^ 0.70–0.74, RPD 1.8–1.9). The calibration for total Mn appeared to be unsuccessful (R^2^ 0.69, r^2^ 0.45, RPD 1.4).

In literature, the information about the applicability of NIRS to accurately measure alkali metals, alkaline earth metals, heavy metals and several non-metals is highly arbitrary. Malley and Williams [30] and Malley et al. [44] reported high prediction accuracy for Ca, K, Mg, Na, P, S, Mn, Fe and Zn with R^2^ values between 0.86 and 0.97, and very high RPD values. However, these promising results might have overestimated the true potential of the NIRS technique to measure these elements, as the distributions of the observed element concentrations were strongly uneven with many close to zero values, which can lead to erroneously high R^2^ values. Bellon-Maurel et al. [36] therefore proposed an index based on inter-quartile distances (RPIQ – ratio of the interquartile distance of reference values to SEP) to prevent this overestimation of calibration qualities due to log-normal distributed variables, which are common in soil science. For our data set, this novel index did not change the general evaluation about whether element concentrations can be measured by means of NIRS or not. Mostly the RPIQs were markedly higher than the RPDs (see Table 2), suggesting a higher potential of the NIRS technique. As most of the element concentrations were relatively evenly distributed across the range of observed values, in our case the RPD value seemed to be the more reliable measure to evaluate calibration quality. Even for the calibration model of total S, where the sample set was divided in two groups of high and low concentrations (see Fig. 2), the RPD appeared to be the more conservative and reliable evaluation measure (see Table 2). To improve prediction accuracy of total S, we tested local calibrations comprising either low or high values. For the sample set with S concentrations below 6.5 g kg^−1^, the R^2^, SEC and SECV were 0.74, 0.66 g kg^−1^ and 0.86 g kg^−1^, respectively. For the sample set with S concentrations above 13.75 g kg^−1^, we obtained R^2^, SEC and SECV values of 0.77, 3.30 g kg^−1^ and 4.07 g kg^−1^. Although R^2^ values were lower compared to the total data set, the prediction errors were significantly reduced, particularly for low-concentrated samples (Table 2). These findings indicate that particularly in cases where sample values show uneven distribution, the development of local calibrations can be an option to improve the prediction quality.

Cozzolino et al. [38] presented accurate NIRS calibrations for total K, Ca, Mg and Fe in soil material across a wide range of soil types, which were roughly in accordance with the results presented here. Contrary, Reeves and Smith [31] concluded that NIR spectroscopic calibrations at a continental scale are generally not possible. However, based on their large data set R^2^ values >0.75 were achieved for Ca and Mg, which do allow at least for approximate quantitative estimations. Poor prediction quality was reported for Al, Fe, K, Na, P, S and Zn. Except for Mn, our results demonstrate that the NIRS technology does have the potential to reliably measure total concentrations of the analyzed elements across a wide range of aquatic sediments. However, care has to be taken at the low ranges, as the predictive power was lower or absent. This suggests at least two preconditions for NIRS measurements: (1) a defined sample population covering a wide range of spectral and chemical characteristics and (2) sufficient high element concentrations [26]. Thus, reliable NIRS measurements of trace metals may be restricted to sediments with high metal contents (e.g. contaminated sites).

### Measurement of NaCl- and oxalate-extractable element concentrations

As for total element concentrations, the performance of NIRS calibrations to predict NaCl-extractable element concentrations was found to be mainly driven by the respective concentrations. Predictions of elements with concentrations <100 mg kg^−1^ appeared to be unsuccessful (Al, Fe, Mn, N-NO_3_-, P, Zn; R^2^ or r^2^ <0.65 and RPD <1.5). Reliable quantitative predictions were, however, obtained for NaCl-extractable Ca, K, Mg, and N-NH_4_ (R^2^ or r^2^ >0.75 and RPD >2.0), which are important target ions for which this extraction technique is applied (cations adsorbed at sediment cation exchange sites). In addition, calibrations that allow for the differentiation between high and low concentrations within the range of observed values could be developed for NaCl-extractable S and Si.

**Figure 3:**
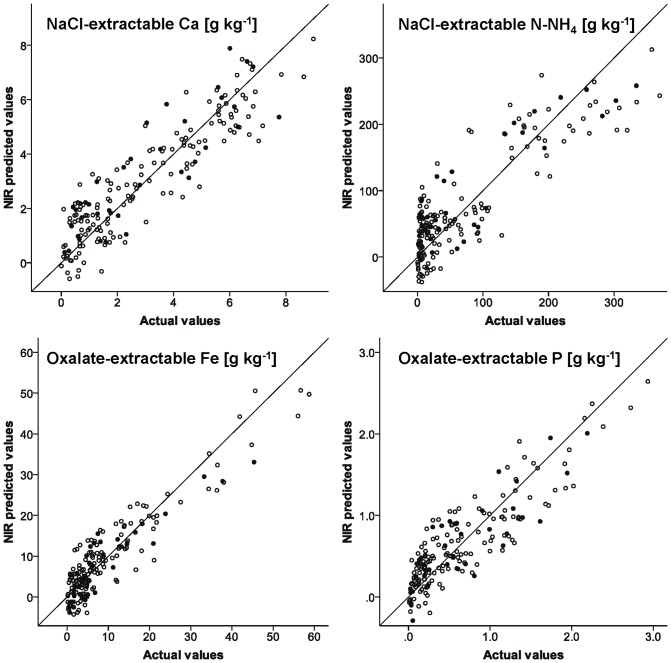
Exemplary observed vs. NIR predicted values for NaCl-extractable Ca and N-NH_4_ and oxalate-extractable Fe and P. *Open circles* calibration data set, *filled circles* external validation.

For oxalate-extractable element fractions, good calibration models were obtained for Al and Fe, which is important to estimate the amount of amorphous Fe and Al available for P binding in the sediment (see Table 2 and Fig. 3). The prediction model for oxalate-extractable P revealed the possibility to distinguish between high and low concentrations within the range of observed values (RPD 1.9; R^2^ and r^2^ 0.78 and 0.72, respectively). Predictions of oxalate-extractable Mn concentrations, however, proved to be unsuccessful. Remarkably, for oxalate-extractable Al, Fe and P acceptable NIRS prediction models could be developed, whereas this was not the case for NaCl-extractable fractions of these elements. This can be explained by the fact that the amorphic Al and Fe fractions are not related to cation exchange sites in the sediment, and therefore not extracted by a NaCl solution. Thus, threshold levels that determine the analytical limit of the NIRS technique strongly depend on the type of extractant, as related to the different sediment fraction involved [19], [45].

To our knowledge NaCl- and oxalate-extractable element fractions in soil or sediment samples have never been measured before by means of NIRS so far. Therefore, direct comparisons to prediction models reported in literature are not possible. However, Chang et al. [19] presented NIRS calibrations for Mehlich III exchangeable metals and exchangeable cations (ammonium acetate extraction). According to the criteria applied here, reliable quantitative prediction models could only be developed for Mehlich III extractable Ca. Model quality was sufficient to distinguish between high and low values for Mehlich III extractable Mg and Mn and Ca and Mg extracted with ammonium acetate, whereas predictions were unsuccessful for the other tested elements (Mehlich III: Cu, Fe, K, P, Zn; ammonium acetate: Na, K). Better performance results for NIRS calibrations to measure exchangeable Ca, Mg and K were obtained by Zornoza et al. [46] and Chodak et al. [47]. This was partly related to strong correlations with the organic matter content. To some extent, correlations to the organic matter content might also be responsible for the good performance of the NIRS models to measure NaCl-extractable Ca and Mg (r>0.7). However, accurate prediction models with RPD values >2.0 could also be developed for extractable element fractions that are hardly related to the organic matter content, e.g. oxalate-extractable Fe (see Fig. 3), whereas predictions were unsuccessful for e.g. NaCl-extractable N-NO_3_ (r = 0.42, see Table 2).

## Conclusions

The presented results show that there indeed is a great potential for NIRS as an analytical tool to improve the understanding of the large spatial variation of sediment characteristics in aquatic systems. Depending on the calibrations available, NIRS offers a rapid and cost-effective alternative to measure multiple important sediment parameters simultaneously. Within less than two minutes, which is the time needed to record a NIR spectrum, various chemical sediment characteristics can be determined. This would otherwise have required different, more labor intensive and costly sample preparation techniques and regular chemical analyses. Moreover, once a spectrum of a sample has been recorded, NIR spectra can be used to retrospectively predict parameters for which calibration models are gained later. In conclusion, the NIRS technology may be highly relevant for scientific studies covering a large number of sampling locations as well as for long-term monitoring or extensive inventory purposes of sediments, e.g. for ecological restoration programs such as large scale dredging of sediments in eutrophied or contaminated surface waters.

## Supporting Information

Figure S1 Observed vs. NIR predicted values for elements and ions not plotted in the manuscript. *Open circles* calibration data set, *filled circles* external validation.
(TIF)

## References

[1] McClain ME, Boyer EW, Dent CL, Gergel SE, Grimm NB, et al (2003) Biogeochemical hot spots and hot moments at the interface of terrestrial and aquatic ecosystems. Ecosystems 6: 301–312.

[2] Shomar BH, Mueller G, Yahya A (2005) Seasonal variations of chemical composition of water and bottom sediments in the wetland of Wadi Gaza, Gaza Strip. Wetlands Ecology and Management 13: 419–431.

[3] Kelderman P, Drossaert WME, Min Z, Galione LS, Okonkwo LC, et al (2000) Pollution assessment of the canal sediments in the city of Delft (the Netherlands). Water Research 34: 936–944.

[4] Stenberg B, Viscarra Rossel RA, Mouazen AM, Wetterlind J (2010) Visible and Near Infrared Spectroscopy in Soil Science. In: Sparks DL, editor. Advances in Agronomy 107. Burlington: Academic Press. 163–215.

[5] Prego R, Filgueiras AV, Santos-Echeandía J (2008) Temporal and spatial changes of total and labile metal concentration in the surface sediments of the Vigo Ria (NW Iberian Peninsula): Influence of anthropogenic sources. Marine Pollution Bulletin 56: 1031–1042. 18334256 10.1016/j.marpolbul.2008.01.036

[6] Arain MB, Kazi TG, Jamali MK, Jalbani N, Afridi HI, et al (2008) Total dissolved and bioavailable elements in water and sediment samples and their accumulation in *Oreochromis mossambieus* of polluted Manchar Lake. Chemosphere 70: 1845–1856. 17889926 10.1016/j.chemosphere.2007.08.005

[7] van der Molen DT, Boers PCM (1994) Influence of internal loading on phosphorus concentration in shallow lakes before and after reduction of the external loading. Hydrobiologia 275: 379–389.

[8] Geurts JJM, Smolders AJP, Banach AM, van de Graaf JPM, Roelofs JGM, et al (2010) The interaction between decomposition, net N and P mineralization and their mobilization to the surface water in fens. Water Research 44: 3487–3495. 20392472 10.1016/j.watres.2010.03.030

[9] Santos-Echeandía J, Prego R, Cobelo-García A, Millward GE (2009) Porewater geochemistry in a Galician Ria (NW Iberian Peninsula): Implications for benthic fluxes of dissolved trace elements (Co, Cu, Ni, Pb, V, Zn). Marine Chemistry 117: 77–87.

[10] Smolders AJP, Lamers LPM, Lucassen E, Van der Velde G, Roelofs JGM (2006) Internal eutrophication: How it works and what to do about it – a review. Chemistry and Ecology 22: 93–111.

[11] Geurts JJM, Smolders AJP, Verhoeven JTA, Roelofs JGM, Lamers LPM (2008) Sediment Fe : PO(4) ratio as a diagnostic and prognostic tool for the restoration of macrophyte biodiversity in fen waters. Freshwater Biology 53: 2101–2116.

[12] Lamers LPM, Smolders AJP, Roelofs JGM (2002) The restoration of fens in the Netherlands. Hydrobiologia 478: 107–130.

[13] Eggleton J, Thomas KV (2004) A review of factors affecting the release and bioavailability of contaminants during sediment disturbance events. Environment International 30: 973–980. 15196845 10.1016/j.envint.2004.03.001

[14] Lucassen E, Smolders AJP, Roelofs JGM (2002) Potential sensitivity of mires to drought, acidification and mobilisation of heavy metals: the sediment S/(Ca+Mg) ratio as diagnostic tool. Environmental Pollution 120: 635–646. 12442787 10.1016/s0269-7491(02)00190-2

[15] Foley WJ, McIlwee A, Lawler I, Aragones L, Woolnough AP, et al (1998) Ecological applications of near infrared reflectance spectroscopy a tool for rapid, cost-effective prediction of the composition of plant and animal tissues and aspects of animal performance. Oecologia 116: 293–305. 28308060 10.1007/s004420050591

[16] Jr. Workman JJ (2008) NIR spectroscopyCalibration Basics. In: Burns DA, Ciurczak EW, editors. Handbook of Near-Infrared Analysis. Boca Raton: CRC Press. 123–150.

[17] Clark DH, Mayland HF, Lamb RC (1987) Mineral analysis of forages with near-infrared reflectance spectroscopy. Agronomy Journal 79: 485–490.

[18] Ritchie GE, Roller RW, Ciurczak EW, Mark H, Tso C, et al (2002) Validation of a near-infrared transmission spectroscopic procedure Part B: Application to alternate content uniformity and release assay methods for pharmaceutical solid dosage forms. Journal of Pharmaceutical and Biomedical Analysis 29: 159–171. 12062675 10.1016/s0731-7085(02)00010-9

[19] Chang CW, Laird DA, Mausbach MJ, Hurburgh CR (2001) Near-infrared reflectance spectroscopy-principal components regression analyses of soil properties. Soil Science Society of America Journal 65: 480–490.

[20] Fuentes M, Hidalgo C, Gonzalez-Martin I, Hernandez-Hierro JM, Govaerts B, et al (2012) NIR Spectroscopy: An Alternative for Soil Analysis. Communications in Soil Science and Plant Analysis 43: 346–356.

[21] Klaus V, Kleinebecker T, Boch S, Müller J, Socher S, et al (2012) NIRS meets Ellenberg's indicator values: prediction of moisture and nitrogen values of agricultural grassland vegetation by means of near-infrared spectral characteristics. Ecological Indicators 14: 82–86.

[22] Kleinebecker T, Schmidt SR, Fritz C, Smolders AJP, Hölzel N (2009) Prediction of δ^13^ C and δ^15^ N in plant tissues with near-infrared reflectance spectroscopy. New Phytologist 184: 732–739. 19691675 10.1111/j.1469-8137.2009.02995.x

[23] Sørensen LK, Dalsgaard S (2005) Determination of clay and other soil properties by near infrared spectroscopy. Soil Science Society of America Journal 69: 159–167.

[24] Kleinebecker T, Klaus VH, Hoelzel N (2011) Reducing sample quantity and maintaining high prediction quality of grassland biomass properties with near infrared reflectance spectroscopy. Journal of near Infrared Spectroscopy 19: 495–505.

[25] Bellon-Maurel V, McBratney A (2011) Near-infrared (NIR) and mid-infrared (MIR) spectroscopic techniques for assessing the amount of carbon stock in soils – Critical review and research perspectives. Soil Biology & Biochemistry 43: 1398–1410.

[26] Chodak M (2008) Application of Near Infrared Spectroscopy for Analysis of Soils, Litter and Plant Materials. Polish Journal of Environmental Studies 17: 631–642.

[27] Shenk JS Jr, Workman JJ, Westerhaus MO (2008) Application of NIR spectroscopy to agricultural products. In: Burns DA, Ciurczak EW, editors. Handbook of Near-Infrared Analysis. New York: Marcel Dekker Inc. 383–431.

[28] Chang CW, Laird DA (2002) Near-infrared reflectance spectroscopic analysis of soil C and N. Soil Science. 167: 110–116.

[29] Terhoeven-Urselmans T, Michel K, Helfrich M, Flessa H, Ludwig B (2006) Near-infrared spectroscopy can predict the composition of organic matter in soil and litter. Journal of Plant Nutrition and Soil Science 169: 168–174.

[30] Malley DF, Williams PC (1997) Use of near-infrared reflectance spectroscopy in prediction of heavy metals in freshwater sediment by their association with organic matter. Environmental Science & Technology 31: 3461–3467.

[31] Reeves JB, Smith DB (2009) The potential of mid- and near-infrared diffuse reflectance spectroscopy for determining major- and trace-element concentrations in soils from a geochemical survey of North America. Applied Geochemistry 24: 1472–1481.

[32] Rossel RAV, Walvoort DJJ, McBratney AB, Janik LJ, Skjemstad JO (2006) Visible, near infrared, mid infrared or combined diffuse reflectance spectroscopy for simultaneous assessment of various soil properties. Geoderma 131: 59–75.

[33] Schwertmann U (1964) Differenzierung der Eisenoxide des Bodens durch photochemische Extraction mit saurer Ammoniumoxalat-Loesung. Zeitschrift für Pflanzenernährung, Düngung und Bodenkunde 105: 194–202.

[34] Houdijk ALFM, Smolders AJP, Roelofs JGM (1993) Effects of atmospheric deposition on the mineral balance in the soil of coniferous forests. Environmental Pollution 80: 73–78. 15091875 10.1016/0269-7491(93)90012-d

[35] Tomassen HBM, Smolders AJP, Lamers LPM, Roelofs JGM (2003) Stimulated growth of *Betula bubescens* and *Molinia caerulea* on ombrotrophic bogs: role of high levels of atmospheric nitrogen deposition. Journal of Ecology 91: 357–370.

[36] Bellon-Maurel V, Fernandez-Ahumada E, Palagos B, Roger J-M, McBratney A (2010) Critical review of chemometric indicators commonly used for assessing the quality of the prediction of soil attributes by NIR spectroscopy. Trac-Trends in Analytical Chemistry 29: 1073–1081.

[37] Saeys W, Mouazen AM, Ramon H (2005) Potential for onsite and online analysis of pig manure using visible and near infrared reflectance spectroscopy. Biosystems Engineering 91: 393–402.

[38] Cozzolino D, Moron A (2003) The potential of near-infrared reflectance spectroscopy to analyse soil chemical and physical characteristics. Journal of Agricultural Science 140: 65–71.

[39] Fidencio PH, Poppi RJ, de Andrade JC, Cantarella H (2002) Determination of organic matter in soil using near-infrared spectroscopy and partial least squares regression. Communications in Soil Science and Plant Analysis 33: 1607–1615.

[40] Brunet D, Barthes BG, Chotte J-L, Feller C (2007) Determination of carbon and nitrogen contents in Alfisols, Oxisols and Ultisols from Africa and Brazil using NIRS analysis: Effects of sample grinding and set heterogeneity. Geoderma 139: 106–117.

[41] Malley DF (1998) Near-infrared spectroscopy as a potential method for routine sediment analysis to improve rapidity and efficiency. Water Science and Technology 37: 181–188.

[42] Chang CW, You CF, Huang CY, Lee TQ (2005) Rapid determination of chemical and physical properties in marine sediments using a near-infrared reflectance spectroscopic technique. Applied Geochemistry 20: 1637–1647.

[43] Malley DF, Lawrence SG, Holoka MH, Williams PC (1996) Applying near-infrared reflectance spectroscopy to predict carbon, nitrogen, phosphorus, and organic-bound cadmium in lake picoplankton. Journal of Aquatic Ecosystem Health 5: 135–147.

[44] Malley DF, Williams PC, Stainton MP (1996) Rapid measurement of suspended C, N, and P from precambrian shield lakes using near-infrared reflectance spectroscopy. Water Research 30: 1325–1332.

[45] Moron A, Cozzolino D (2007) Measurement of phosphorus in soils by near infrared reflectance spectroscopy: Effect of reference method on calibration. Communications in Soil Science and Plant Analysis 38: 1965–1974.

[46] Zornoza R, Guerrero C, Mataix-Solera J, Scow KM, Arcenegui V, et al (2008) Near infrared spectroscopy for determination of various physical, chemical and biochemical properties in Mediterranean soils. Soil Biology & Biochemistry 40: 1923–1930. 23226882 10.1016/j.soilbio.2008.04.003 PMC3517214

[47] Chodak M, Khanna P, Horvath B, Beese F (2004) Near infrared spectroscopy for determination of total and exchangeable cations in geologically heterogeneous forest soils. Journal of near Infrared Spectroscopy 12: 315–324.

